# Involvement of Alfin-Like Transcription Factors in Plant Development and Stress Response

**DOI:** 10.3390/genes15020184

**Published:** 2024-01-29

**Authors:** Ruixin Jin, Haitao Yang, Tayeb Muhammad, Xin Li, Diliaremu Tuerdiyusufu, Baike Wang, Juan Wang

**Affiliations:** 1 Key Laboratory of Genome Research and Genetic Improvement of Xinjiang Characteristic Fruits and Vegetables, Institute of Horticulture Crops, Xinjiang Academy of Agricultural Sciences, Urumqi 830091, China; 18099304389@163.com (R.J.); yht18893912614@163.com (H.Y.); tayebmuhammad1@hotmail.com (T.M.); lxinlx1998@163.com (X.L.); 320223347@xjau.edu.cn (D.T.); 2 College of Life Science and Technology, Xinjiang University, Urumqi 830046, China; 3 College of Computer and Information Engineering, Xinjiang Agricultural University, Urumqi 830052, China

**Keywords:** Alfin-like transcription factors, structural characteristics, growth and development, abiotic stress

## Abstract

Alfin-like (AL) proteins are an important class of transcription factor (TF) widely distributed in eukaryotes and play vital roles in many aspects of plant growth and development. AL proteins contain an Alfin-like domain and a specific PHD-finger structure domain at the N-terminus and C-terminus, respectively. The PHD domain can bind to a specific (C/A) CAC element in the promoter region and affect plant growth and development by regulating the expression of functional genes. This review describes a variety of AL transcription factors that have been isolated and characterized in *Arabidopsis thaliana*, *Brassica rapa*, *Zea mays*, *Brassica oleracea*, *Solanum lycopersicum*, *Populus trichocarpa*, *Pyrus bretschenedri*, *Malus domestica*, and other species. These studies have focused mainly on plant growth and development, different abiotic stress responses, different hormonal stress responses, and stress responses after exposure to pathogenic bacteria. However, studies on the molecular functional mechanisms of Alfin-like transcription factors and the interactions between different signaling pathways are rare. In this review, we performed phylogenetic analysis, cluster analysis, and motif analysis based on *A. thaliana* sequences. We summarize the structural characteristics of AL transcription factors in different plant species and the diverse functions of AL transcription factors in plant development and stress regulation responses. The aim of this study was to provide a reference for further application of the functions and mechanisms of action of the AL protein family in plants.

## 1. Introduction

During the plant growth cycle, plants face various unfavorable conditions in the form of biotic and abiotic stress. Abiotic stress such as drought, salinity, and high and low temperatures are adverse environmental conditions that affect plant growth and development and ultimately reduce final crop production [1,2]. Under different stress conditions, plants modify or adapt different metabolic processes related to genetics or physiology [3,4]. Many important biological processes are associated with the regulation of gene expression. These regulatory pathways are complex and diverse and are influenced by a variety of factors, including transcription factors, which are now being studied in increasing detail for their effects on plant growth and development.

Transcription factors (TFs) are a class of regulatory proteins that bind to specific sequences upstream of the 5′ end of target genes and play a critical role in the translation of stress signal perception into stress-responsive gene expression. During signal transduction, transcription factors interact with *cis*-acting elements in the promoter regions of various stress-responsive target genes, thereby activating gene signaling cascades that act together and enhance plant tolerance to different environmental stresses [5]. To date, a number of stress-related transcription factors, including *DREB*, *NAC*, *MYB*/*MYC*, *WRKY*, *bZIP*, Alfin-like (AL) genes, and several other families, have been identified in plants [6,7,8,9,10,11]. These different families of transcription factors respond to various biotic and abiotic stresses in plants. Most plants are susceptible to biotic and abiotic stressors that affect yield and survival throughout their life cycle. Plants have developed tolerance systems that modify cellular biochemistry and physiology by altering gene expression in response to stress [12,13,14]. Among the TFs, stress-responsive TFs are involved in increasing plant adaptation to adverse environmental stress, such as cold, salt, and drought, as well as defense responses against invading pathogens [4,15]. The activity of these transacting factors represents a dominant and dynamic mechanism by which higher terrestrial plants can adapt to their environment [16].

AL transcription factors are a class of specific transcription factors involved in various types of stress in plants. It has been reported that the AL gene family plays crucial roles in plant growth and development, such as seed germination, root development, root hair elongation, and meristem development [17,18,19]. There are two main structural domains present in all AL proteins: an N-terminal conserved structural domain consisting of 140 conserved amino acid residues (the Alfin structural domain) and a C-terminal conserved PHD-finger structural domain consisting of 50 conserved amino acid residues (the Cys4-His-Cys3 type), along with a variable region, the V structural domain, located between these two structural domains [20]. Both of these particular structural domains play important roles in plants and in promoting different stress responses to different environments.

Due to the important functions and limited reports of AL transcription factors in plants, the main purpose of this review was to summarize and collate the results of other studies on AL proteins and to perform simple bioinformatics analysis of AL proteins based on the results of several years of related research. For this purpose, a phylogenetic tree of the AL gene family was constructed, and cluster analysis, motif analysis, and corresponding function analysis of AL proteins were performed. In addition, we also provide an outlook for future research in the hopes that further studies on the molecular mechanism and different signal transduction pathways of this gene family can be carried out in the future. This review will lay the foundation for revealing the uncharacterized functions of the relevant AL proteins in plants.

## 2. Survey Methodology

Based on previous research reports on AL proteins in *A. thaliana* (https://www.arabidopsis.org/, accessed on 20 September 2023), *Z. mays* (https://www.maizegdb.org/, accessed on 20 September 2023), *B. rapa* and *B. oleracea* (https://brassicadb.cn, accessed on 20 September 2023), *Oryza sativa* (https://plants.ensembl.org/index.html, accessed on 20 September 2023), *S. lycopersicum* (https://solgenomics.net, accessed on 20 September 2023), and *P. trichocarpa* (https://plants.ensembl.org/index.html, accessed on 10 January 2024), we searched for AL proteins in different species by reviewing and analyzing the contents of the relevant literature, including the earliest analyses of *A. thaliana* and relevant articles that have been published within the last few years. All relevant protein sequences were described in previous related studies. The different genome sequences were used to construct a phylogenetic tree using MEGA11 according to the LG + F method. Gene structures were mapped using TBtools based on relevant annotation information, and conserved structural domains were obtained using HMMER (https://www.ebi.ac.uk/Tools/hmmer/search/hmmscan, accessed on 13 January 2024). The conserved protein motifs of the AL transcription factors from seven species were analyzed using MEME (https://meme-suite.org/meme/tools/meme, accessed on 13 January 2024). Figure quality was improved using version 2020 of Adobe Illustrator software (Adobe Illustrator 2020.lnk, accessed on 13 January 2024).

## 3. Structural Characteristics and Classification of the Alfin-Like Transcription Factors

Alfin-like transcription factors are small and unique transcription factors in plants that play different roles at different stages of growth and development. Alfin-like transcription factors were first identified in *Medicago sativa* (*alfalfa*). In that study, Alfin was revealed to be the first storage protein synthesized during the development of somatic cells and syncytial embryos. In somatic embryos, the predominance of Alfin 7S storage proteins is associated with an increase in their mRNA accumulation. Mature (14 d) somatic embryos were closest to stage VI syncytial embryos in terms of protein and mRNA accumulation. Developmental comparisons also indicate that the synthesis patterns of individual storage proteins are regulated independently of each other during *alfalfa* embryogenesis [21]. AL proteins also showed 50–66% sequence identity to *alfalfa Alfin1* [20]. Previous studies reported that AL proteins are mainly localized in the nucleus and play crucial roles in plant transcriptional regulation [11,20,22,23]. AL transcription factors are two-domain proteins that can bind to highly methylated forms of histones and function in plant abiotic stress [23,24]. AL proteins have two conserved domains located at the N-terminus and C-terminus, namely, the DUF3594 domain (or Alfin domain) and the PHD-finger domain. In addition to shaping the stress response, reactive oxygen species (ROS) act as signaling mediators to maintain redox homeostasis. Recent studies have shown that reactive oxygen species production and redox metabolism are closely related to endoplasmic reticulum stress. The reactive oxygen species (ROS)-related transcription factor *GmAlfin09* and the peroxidase *GmPRDX6* have been identified in soybeans, and experimental results confirm that *GmAlfin09* promotes the up-regulation of *GmPRDX6*, which reduces ROS levels and promotes endoplasmic reticulum stability [25]. Although the DUF3594 domain has been less well studied, the highly conserved nature of the DUF3594 domain across species suggests that AL proteins may have a fundamental biological function in plants. The AL family is a plant-specific subfamily of plant homeodomain (PHD) finger proteins [22]. Similarly, PHD-finger proteins exhibit root-specific salt responses after binding to conserved GNGGTG or GTGGNG sites in the promoter regions of target genes [26]. PHD-finger proteins play crucial roles in different physiological processes in plants. For example, it mediates epigenetic silencing and vernalization-induced flower regulation in *Arabidopsis* [23,27,28,29]; it also binds to histone H3K4me3/2 [23] and to *OBERON1* (OBE1) and *OBERON2* (OBE2) to maintain root tip meristematic tissue [30]. In soybean, the histone methylation and acetylation of different lysine residues provide a platform for *GmPHD* to bind to stress-related genes and regulate their expression under salt stress [31]. The N-terminal DUF3594 structural domain (also known as the PAL structural domain) [32] consists of 140 highly conserved amino acids that functionally mark the transcriptional start site of all genes and recognize trimethylation of Lys-4 of histone H3 (H3K4me3) [18,33,34]. This structural domain contains five α-helix structures and two β-structures and exists as a conserved homodimer of AL family proteins [32]. However, no AL proteins containing this structural domain have been reported in animals, fungi, or prokaryotes [35].

**Figure 1:**
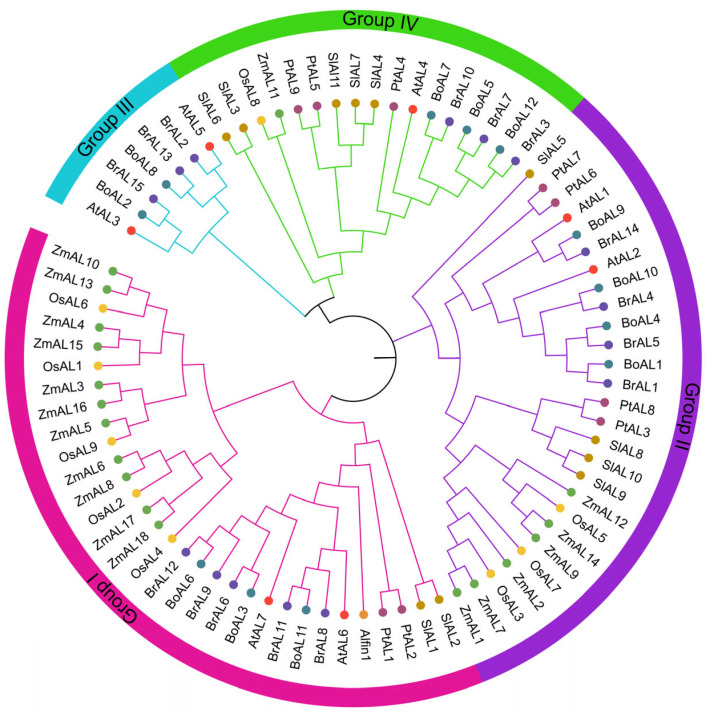
Phylogenetic analysis of *Arabidopsis thaliana*, *Brassica rapa*, *Zea mays*, *Brassica oleracea*, *Solanum lycopersicum*, *Populus trichocarpa* and *Oryza sativa* AL transcription factors. The graph was constructed in MEGA11 using the LG + F approach.

Previously, several AL transcription factors were identified in different crop species, such as *A. thaliana*, *B. rapa*, *B. oleracea*, *Z. mays*, *S. lycopersicum*, *O. sativa*, *P. trichocarpa***,** *P. bretschenedri*, and *M. domestica*, which contained 7, 15, 12, 18, 11, 9, 9, 15 and 11 AL members, respectively [11,14,36,37,38,39,40,41]. To better understand the evolutionary relationships of AL TFs, based on the similarity of protein sequences, an unrooted phylogenetic tree was constructed using the LG + F method. According to the phylogenetic tree, the AL protein family members of seven species were divided into four branches (Figure 1), which is consistent with the previously reported *A. thaliana* AL protein classification [36]. Interestingly, the distribution of the AL genes varied among the groups based on the species; for example, almost all the maize AL genes were clustered into groups one and two, with the third group containing the fewest members and no members from maize. Nine *PtAL* genes were distributed across all groups except for the third group, and they exhibited different levels of stress response to salt, drought, cold, and heat [39]. This difference might be due to the high sequence similarity between the different maize AL proteins. The various AL genes clustered in different groups perform specific functions. *AtAL5*, in the third group, regulates various signaling pathways in plants, and overexpression of *AtAL5* repressed the transcription of downstream negative regulatory genes, including *SHMT7*, *TAC1*, *OFE*, *FAO*, and *CAX1*, to improve salt, drought, and cold stress tolerance in transgenic seedlings [20]. *BoAL8* is responsive to different abiotic stresses [38]. It has also been suggested that the novel Cys4-His-Cys3 potential zinc finger structure is distributed within group one of *alfalfa* proteins; *Alfin1* might play a role in transcriptional regulation; and *Alfin1* might bind to G-rich DNA elements in the promoter of *MsPRP2*, a salt-inducible and root-specific gene [26]. *AL6* in *A. thaliana* responds differently to phosphorus-stimulated elongation of root hairs and *AL7* to salt stress [17,36]. *BoAL12* and *SlAL3*, in the fourth group, also exhibit some stress responses to different abiotic stresses. In addition, AL proteins can bind to PRC1 to form complexes and thus participate in many complex biological processes. For example, the SUMO protease *FUG1* physically interacts with *AL3*, and interference with its potential SUMOylation site disrupts its nuclear localization. *AL3* interacts with LHP1 of the PRC1 complex, and the FUG1-AL3-LHP1 module is essential for conferring repeat amplification-associated epigenetic silencing of the SUMO protease *FUG1*, histone reader *AL3*, and PRC1 complex, which are integral to repeat expansion-induced epigenetic silencing in *A. thaliana* [42].

**Figure 2:**
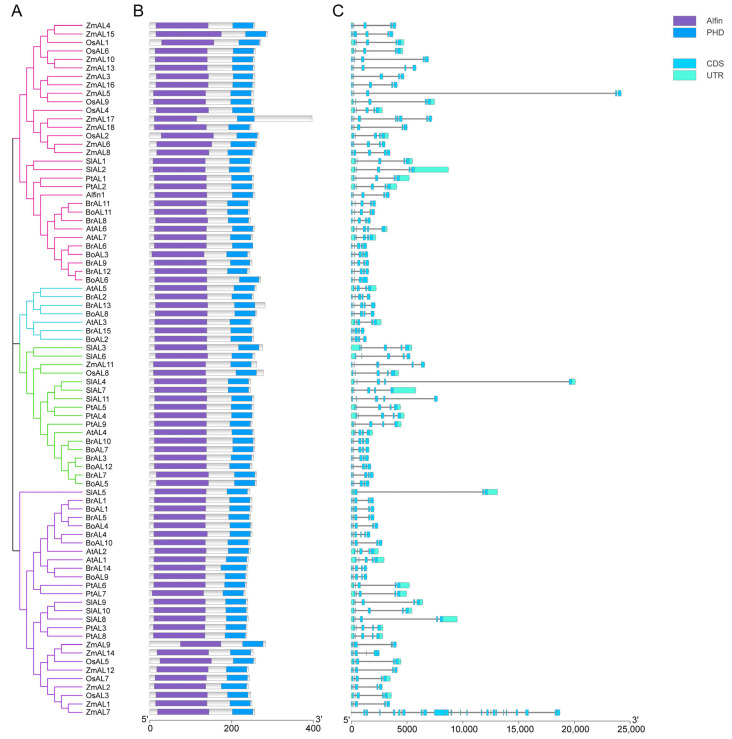
Cluster analysis of *A. thaliana, Z. mays, B. oleracea, B. rapa, S. lycopersicum, O. sativa,* and *P. trichocarpa* AL transcription factors. (**A**): Phylogenetic tree of 82 AL transcription factor proteins; (**B**): Conserved structural domains. Purple for Alfin structure, blue for PHD structure; (**C**): Gene structure. CDS and UTR are shown in different coolers, and introns are represented by a black line.

**Figure 3:**
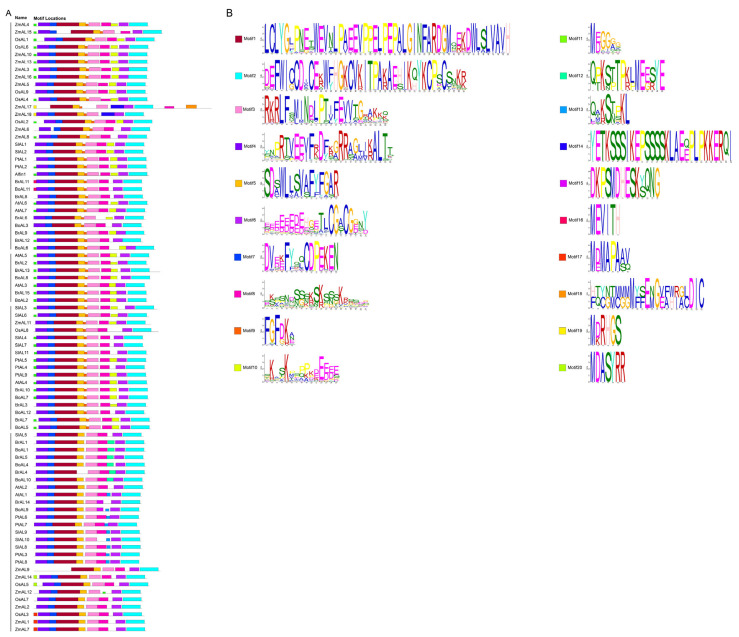
Motif analysis of *A. thaliana, Z. mays, B. oleracea, B. rapa, S. lycopersicum, O. sativa,* and *P. trichocarpa* AL transcription factors (**A**): Structure of 82 AL transcription factor protein conserved motifs. Different color boxes represent different motifs; (**B**): 20 motifs. Protein conserved motifs were analyzed using MEME (Introduction-MEME Suite) (https://meme-suite.org/meme/tools/meme, accessed on 13 January 2024) for AL transcription factors in seven species.

Similarly, cluster and conserved motif analyses revealed that the AL family proteins contained two conserved domains, the Alfin domain and the PHD domain (Figure 2B). The PHD-finger and Alfin domain distribution sites indicated that these two domains had coevolutionary connections with one another. To gain insight into the diversity of the gene structures, we performed a simple structural analysis of the full-length cDNA of AL genes (Figure 2C). Structural analyses revealed that most of the genes with similar numbers and lengths of introns/exons were typically clustered into the same group. The AL proteins distributed in different groups are essentially composed of 5–6 CDSs, and only individual CDSs differ in number. The lengths of the introns are different across all AL members but somewhat similar within the same group. In general, similarities in the gene structure are closely mirrored in the phylogenetic tree. Motif analysis revealed that almost all the AL proteins in the different species contained the two essential structural domain motifs. Among them, motifs 2 and 6 encode the PHD-finger structural domain, and motifs 1, 3, and 5 encode the DUF3594 structural domain (Figure 3A). Motifs 4 and 7 are absent only in *ZmAL17* and *ZmAL9*, while both motif structures are present in all the other proteins. Motif 8 is present in most AL proteins, and a small number of AL members do not have this structure. We speculate that this difference may be related to later evolution. None of the 27 AL proteins clustered in the second group had motif 9, which was present in members of the other three groups. We speculate that this pattern is related to the proximity of evolutionary relationships between species. The fact that all the AL family members with similar exon/intron numbers and motif patterns clustered in the same group implies that they have similar functional roles in plants. However, there is little information about the function of AL transcription factors in plants, and further investigation is required to determine the complete regulatory mechanism involved.

## 4. Role of Alfin-Like Transcription Factors in Plant Growth and Development

### 4.1. Alfin-Like Transcription Factors Regulate Seed Shape and Seed Germination

Rice is one of the most important food crops in the world. Recently, grain-related AL genes have been identified in rice. The *OsAL* genes were analyzed and found to be localized in the nucleus and not to have transcriptional self-activating activity. Most of the natural variants of *OsALs* were significantly associated with yield traits, grain size differences, and drought tolerance. It was observed that the *OsAL7.1* and *OsAL11* transgenic plants exhibited significantly regulated seed shape under drought stress and were more sensitive to ABA and mannitol at the germination stage [43]. The PAL domain at the N-terminus of AL proteins can bind to the PRC ring finger and participate in the seed germination process, and the function of AL PHD-PRC1 complexes is to convert the H3K4me3 active state to the H3K4me3 repressive state of seed developmental genes during seed germination. T-DNA insertion mutant analysis revealed that simultaneous loss of *AtAL6*/*AtAL7* and *AtBMI1a*/*AtBMI1b* retards seed germination due to loss of function of the AL PHD-PRC1 complex [19].

### 4.2. Alfin-Like Transcription Factors Regulate Plant Root Development

Phosphorus deficiency is a limiting factor for plant growth, and a limited amount of plant-available soil phosphorous leads to severe yield losses [44]. Phosphorus deficiency causes a decrease in the number of primary roots and an increase in lateral root density and length [45,46,47,48]. Alfin-like transcription factor AL6 pure mutants produced shorter root hairs under phosphorus deficiency conditions, probably caused by altered expression of its downstream target genes. In addition to affecting root hair length, the per2 mutant also exhibited changes in primary root elongation, lateral root numbers, anthocyanin accumulation, and phosphorous ion concentrations. These results suggest that AL6 acts as a novel upstream regulator of root hair formation during phosphorus deficiency in A. thaliana [17]. Overall, we speculate that there are likely to be growth- and development-related functions of AL transcription factors, but confirmation of this speculation requires additional work in the future.

## 5. Role of Alfin-Like Transcription Factors in Plant Biotic and Abiotic Stress

### 5.1. Role of Alfin-Like Transcription Factors in Biotic Stress

Biotic stress, such as those applied by insects, pathogens, and weeds, affects plant morphological, physiological, and metabolic processes as well as defense responses [49,50,51]. Maize anthracnose, caused by fungal infiltration, severely compromises maize yield. In maize leaves infected by Gramineae anthracnose, *ZmAL5a* expression continuously increased with time. It has been shown that inoculation of plant leaves with B. graminids activates the resistance of distal uninoculated plant tissues to fungal pathogens and that this signaling involves the accumulation of β-1,3-glucanase in SA and abscisic acid (ABA) signaling. Gene expression analysis of ZmAL5a in the C_4_ crop maize further showed that the ZmAL5a, *β-1,* and *3-GA2* genes not only respond to abiotic stress (salt and drought) but also that *ZmAL5a* is involved in the signaling pathway for *β-1,3-GA2* gene expression [52]. B. oleracea contains 12 ALs transcription factors, and it was observed that various abiotic and biotic stress significantly enhanced the transcript levels of these ALs, particularly the expression of BoAL8 and BoAL12, after inoculation with Pectobacterium carotovorum subsp. carotovorum [38]. The Vitis quinquangularis AL transcription factor VqAL4 binds directly to the G-rich element (CACCTC) promoter of VqNSTS4 to activate its expression. In addition, overexpression of VqAL4 positively regulates powdery mildew resistance in grapes by inducing stilbene accumulation and SA signaling [53]. Recent studies have shown that in tobacco mosaic virus (TMV)-infected cells, the NLR protein induces phosphorylation of the transcription factor AL7 through activation of the mitogen-activated protein kinase (MAPK) cascade. AL7 suppressed downstream ROS-scavenging genes and enhanced ROS accumulation in the infected cells. However, under normal conditions, NLR proteins inhibit AL7 phosphorylation to prevent the down-regulation of ROS-scavenging genes and excess ROS accumulation [54].

### 5.2. Role of Alfin-Like Transcription Factors in Abiotic Stress

To date, studies on AL transcription factors are few. In 1992, the *Alfin1* gene was first identified in *alfalfa*, and its overexpression was induced by salt stress [21]. A total of 7 *AL* genes were identified in *A. thaliana* [55], and *Thellungiella halophila ALs* have a high degree of sequence identity with those of *A. thaliana*. In plants, *AL* genes regulate multiple signaling pathways related to environmental factors and abiotic stress tolerance [26,36]. In addition, four *AhAL* genes were isolated in *Atriplex hortensis*, all of which bind to *cis*-acting and trans-inhibitory nuclear localization proteins. Previously, *AhAL1*-expressing transgenic plants were shown to have greater survival under salt and drought stress conditions, with lower MDA contents and less water loss than wild-type plants. *AhAL1* can bind to the promoter regions of *GRF7*, *DREB1C*, and multiple groups of *PP2C* genes to inhibit their expression, resulting in abscisic acid (ABA)-mediated stomatal closure, seed germination inhibition, and primary root elongation enhancement in transgenic plants. However, the expression levels of the stress-related positive regulatory genes *DREB1A*, *DREB2A*, and *ABFs* were increased in *AhAL1* overexpressing plants [22]. With the continuous development of bioinformatics, an increasing number of AL transcription factors have been identified in plants. Fifteen *BrALs* were identified in kale, and all of these *BrALs* were induced by cold, salt, and drought stress. Similarly, 18 *ZmAL* genes were identified in maize, and various abiotic stresses increased the transcript levels of many *AL* genes in maize. The AL family comprises nonspecific transcription factors involved in abiotic stress response in dicotyledonous plants, and early studies have shown that the expression of *OsALs* is regulated by different environmental stimuli and phytohormones, with overexpression of *OsAL7.1* and *OsAL11* in rice impairing drought tolerance at the maturity stage [43]. Both ING proteins and AL proteins are involved in chromatin regulation of nuclear proteins through binding to H3K4me3/2. The Alfin proteins are also considered H3K4me3/2 readers [19,23] and perform functions related to *AtRING1* and *AtBMI1* interactions [56]. Six members of the *AL* gene family were identified in grapevine, and analysis of the upstream promoter region revealed a large number of ***cis***-acting elements related to phytohormones and abiotic stress responses. Expression pattern analysis revealed that these AL transcription factors are closely related to the phytohormones ABA, drought, salt, and low-temperature stress. In particular, members of this family strongly respond to salt stress [57].

In recent years, an increasing number of researchers have begun to study AL transcription factors. The *Daucus carota DcPSY2* gene promoter contains Alfin response elements that bind to *DcAL4* and *DcAL7* to regulate various developmental and stress-related regulatory mechanisms. Similarly, the expression of both the *DcAL4* and *DcAL7* genes was induced by ABA treatment and salt stress conditions. Furthermore, the ecotypic expression of *DcAL4* and *DcAL7* enhanced the survival of *A. thaliana* seedlings during salt stress conditions [58]. In apples, all 11 *MdAL* genes exhibited differential expression patterns after exposure to various abiotic stress conditions. Similarly, compared with the wild type, the overexpression of *MdAL4* positively regulates *A. thaliana* seedling tolerance to drought stress by reducing drought stress damage such as from ROS, malondialdehyde (MDA), and electrolyte leakage [41]. Recently, genome-wide identification and analysis of the AL protein family in cultivated tomato (*S. lycopersicum*) and three wild relatives (*S. pennellii*, *S. pimpinellifolium*, and *S. lycopersicoides*) were performed to evaluate the AL response to different abiotic stresses, and it was revealed that nuclear localized *SlAL3* gene expression was induced by both drought and salt stress [11]. Fifteen *PbAL* genes have been studied and reported in *P. bretschenedri*, and analysis of the promoter regions revealed a number of stress-related *cis*-acting elements associated with hormonal and environmental stress responses. The differential expression of *PbAL* genes under indoleacetic acid, gibberellin, melatonin, and abscisic acid treatments was subsequently analyzed via qRT–PCR, which revealed that 15 *PbAL* genes responded to both abiotic stress and fruit growth and development [40]. Nine AL transcription factors were identified in poplar, and analysis of these nine proteins revealed that most of the *PtAL* genes were highly expressed at low temperature (4 °C), at high temperature (39 °C), under 100 mM NaCl treatment, and under drought treatment. *PtAL4* and *PtAL6* are sensitive to different types of stress, and *PtAL5* and *PtAL7* exhibit resistance to different types of stress. *PtAL* genes play important roles in plant growth and development and in response to abiotic stress, providing valuable insights into plant stress tolerance [39].

**Figure 4:**
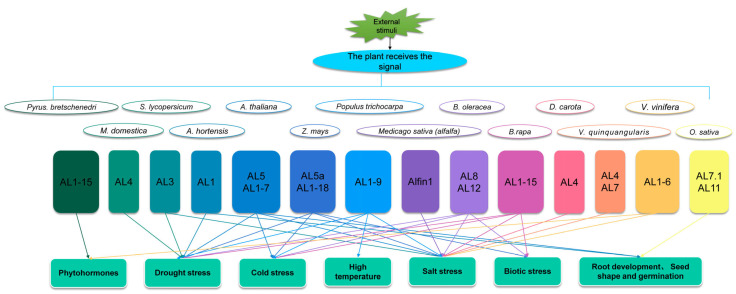
Research progresses on AL transcription factors.

The above studies showed that the *AL* gene family is widely involved in plant growth and development and in response to various biotic and abiotic stresses, as shown in Figure 4 and Table 1.

## 6. Conclusions and Perspectives

Alfin-like proteins play an important role in many aspects of plant growth and development and in response to various stressors. In recent years, researchers have successively cloned and identified members of the *AL* gene family in different plants and performed biological functional analysis. This review is based on genomic data from different species, and this comprehensive analysis as well as the review of previous functions identified by bioinformatics methods can not only help us better understand the functions that these genes are already known to have but also provide more research directions for elucidating the functions of these genes in the future. The involvement of *AL* genes in salt, drought, and cold stress conditions in plants has been extensively reported, and these studies have led to a deeper understanding of the members of the *AL* gene family for future studies. Our analyses revealed that the evolutionary relationships between different species are relatively similar and that proteins grouped in the same phylogenetic group have similar gene structures and functions. Therefore, we hypothesized that most of the genes with unknown functions may also respond to abiotic stress in plants. Alfin-like transcription factors are commonly found in higher plants, and with the development of transcriptome sequencing technology and the updating of whole genome annotations, an increasing number of *AL* genes will be identified or reannotated in different plant species. However, further research is still needed to determine the exact functions of the AL protein. At present, many studies have reported the functional role of the plant *AL* gene family in determining the effects of plant shape, seed germination, root hairs, and abiotic stress. These studies are limited to the identification and initial characterization of the gene family, and the complete molecular mechanisms and signal transduction pathways of *ALs* in plant-specific biological functions have not been thoroughly studied. Recent studies have also revealed that some ALs can regulate different hormonal signals. Therefore, we speculate that these genes not only play a role in one signaling pathway but can also interact with multiple signaling pathways. However, studies on the molecular mechanisms of AL genes are still rare.

In conclusion, an in-depth study of the biological functions of AL proteins and the construction of molecular regulatory networks will enrich and improve our understanding of the roles of AL proteins in various biological events. Furthermore, such research will also provide a valuable scientific basis for the study of new AL proteins. Therefore, identifying additional *AL* gene family members involved in various types of stress and analyzing their mechanisms of action in plants are highly important and valuable. These studies will be highly important for plant stress resistance breeding and genetic characterization.

## Figures and Tables

**Table 1:** Research progresses on AL transcription factors.

Species	Gene	Function	References
*Alfalfa*	Alfin1	salt stress	[18,26,33]
*A.thaliana*	AL5	salt, drought, and cold stress	[20]
	AL7	salt stress	[36]
	AL6	control root hair elongation under phosphate deficient conditions	[17]
*A. hortensis*	AL1	drought stress, mediated ABA pathway, consistent seed germination, reduced initial rooting	[22]
*O.sativa*	AL7.1, AL11	Regulation of seed shape, responds to ABA and mannite	[43]
*Z.mays*	AL5a	salt, drought stress, and fungal pathogens	[52]
	AL1-18	salt, drought, and cold stress	[14]
*B.oleracea*	AL8, AL12	salt, drought, cold stress, and fungal stress	[38]
*B.rapa*	AL1-15	salt, drought, cold stress, and fungal stress	[37]
*M.domestica*	AL4	drought stress	[41]
*S.lycopersicum*	AL3	salt, drought stress	[11]
*D.carota*	AL4, AL7	salt stress	[58]
*V.quinquangularis*	AL4	Promotion of stilbene accumulation, activation of SA signaling, and enhancement of resistance to powdery mildew	[53]
*V.vinifera*	AL1-6	salt stress, phytohormones	[57]
*P. bretschenedri*	AL1-15	response to indole acetic acid, gibberellin, melatonin, and abscisic acid	[40]
*P. trichocarpa*	AL1-9	salt, drought, cold, and high temperature stress	[39]

## Data Availability

Not applicable.
